# Surgical workflow recognition with 3DCNN for Sleeve Gastrectomy

**DOI:** 10.1007/s11548-021-02473-3

**Published:** 2021-08-20

**Authors:** Bokai Zhang, Amer Ghanem, Alexander Simes, Henry Choi, Andrew Yoo

**Affiliations:** C-SATS, Inc. Johnson & Johnson, 1100 Olive Way, Suite 1100, Seattle, WA 98101 USA

**Keywords:** Surgical workflow recognition, Computer-assisted surgery, 3D ConvNet, Focal loss

## Abstract

**Purpose:**

Surgical workflow recognition is a crucial and challenging problem when building a computer-assisted surgery system. Current techniques focus on utilizing a convolutional neural network and a recurrent neural network (CNN–RNN) to solve the surgical workflow recognition problem. In this paper, we attempt to use a deep 3DCNN to solve this problem.

**Methods:**

In order to tackle the surgical workflow recognition problem and the imbalanced data problem, we implement a 3DCNN workflow referred to as I3D-FL-PKF. We utilize focal loss (FL) to train a 3DCNN architecture known as Inflated 3D ConvNet (I3D) for surgical workflow recognition. We use prior knowledge filtering (PKF) to filter the recognition results.

**Results:**

We evaluate our proposed workflow on a large sleeve gastrectomy surgical video dataset. We show that focal loss can help to address the imbalanced data problem. We show that our PKF can be used to generate smoothed prediction results and improve the overall accuracy. We show that the proposed workflow achieves 84.16% frame-level accuracy and reaches a weighted Jaccard score of 0.7327 which outperforms traditional CNN–RNN design.

**Conclusion:**

The proposed workflow can obtain consistent and smooth predictions not only within the surgical phases but also for phase transitions. By utilizing focal loss and prior knowledge filtering, our implementation of deep 3DCNN has great potential to solve surgical workflow recognition problems for clinical practice.

## Introduction

Computer-assisted surgery (CAS) system is one of the cornerstones for modern operating rooms. One essential aspect of building this system is surgical workflow recognition. Surgical workflow recognition can be used to locate the main surgical phases from surgical videos. Video clips that contain main surgical phases can be used in the expert review process, which will help surgeons further develop their skills. Surgical workflow recognition can also be used to calculate the operating time for each surgical phase, which can help surgeons benchmark their performance. Automatic surgical workflow recognition not only provides a tool to understand surgeon performance, but can also enhance coordination among surgical teams, leading to improved surgeon skills and better patient outcomes.

Computer vision-based automatic surgical workflow recognition has gained a lot of attention in recent years. Early research proposes using deep convolution neural networks to classify videos frame-by-frame without using temporal information [1–4]. Other approaches utilize CNN–RNN (convolutional neural network–recurrent neural network) to capture both spatial and temporal information for surgical workflow recognition [5–14]. Typical design choices for CNN in these approaches are ResNet [15] or Inception [16]. These deep convolutional neural networks can capture spatial information for each frame from the surgical video. A typical design choice for RNN is long short-term memory (LSTM) which is used to capture the temporal information between frames from the surgery video [6, 7]. With the rise of 3DCNNs, a shallow 3DCNN design like a C3D network was proposed to solve the surgical workflow recognition problem [17]; however, the shallow C3D network did not outperform the CNN–RNN design.

Instead of using a shallow C3D, we choose a deep architecture known as Inflated 3D ConvNet (I3D) [18] for our workflow. We implement a 3DCNN workflow referred to as I3D-FL-PKF. This combines I3D with focal loss (FL) [19] and prior knowledge filtering (PKF) for surgical workflow recognition. The goal of this workflow is to improve the results obtained by using a CNN–RNN-based architecture, which is commonly used for similar tasks. The rationale behind using a 3DCNN is to capture spatial and temporal information inside surgical videos.

## Dataset and annotation

To test our proposed workflow, we collected robotic and laparoscopic surgical videos for sleeve gastrectomy from 14 institutions. This procedure is used to assist patients with weight loss and reduce the risk of potentially life-threatening weight-related health problems. Sleeve Gastrectomy can be performed for patients who require anti-inflammatory medication or for patients who suffer from conditions such as cirrhosis, anemia, or severe osteoporosis which preclude intestinal bypass [20]. According to the literature [20–24], our medical experts split sleeve gastrectomy procedure into eight surgical phases: “Exploration/inspection,” “Ligation of short gastric vessels,” “Gastric transection,” “Bougie,” “Oversew staple line,” “Liver retraction,” “Hiatal hernia repair,” and “Gastric band removal.” The parts of the video that did not get annotated were named as “Not a phase.” Video segments annotated as “Not a phase” usually are surgical phase transaction segments, undefined surgical phase segments, out-of-body segments, idle segments, and so on. Understanding the above-mentioned surgical phases and locating them in the surgical videos can be valuable for skill assessments. Early research [25] shows that “Ligation of short gastric vessels” and “Oversew staple line” are the two most hazardous surgical phases in sleeve gastrectomy cases, and where the majority of technical errors were made. Video clips that contain these surgical phases can be used in the expert review process to help the surgeon improve on these complex surgical phases. Locating surgical phases that are optional in the procedure, such as “Liver retraction,” can also indicate the need for further clinical research to understand the benefit of completing these phases. This can support standardizing surgical phases.

In this project, 461 videos were gathered and annotated with the above-mentioned set of phases. The framerate for our video is 30 frames per second. The dimensions of our videos are either $768\times 480$ or $854\times 480$. To train the proposed deep learning workflow, 317 videos were used for the training dataset, and 82 videos were used for the validation dataset. A dataset of 62 videos was used to test the workflow after training.

As shown in Table 1, we calculate the hours of video data we have for the training, validation, and test datasets. We have a very imbalanced dataset due to the duration of the surgical phase varies from each other. Another reason that causes this dataset imbalance problem is many surgical phases are optional, for example: “Liver retraction,” “Hiatal hernia repair,” “Bougie,” and “Gastric band removal.” From Table 1, we have a very limited amount of training data for several surgical phases for example: “Liver retraction,” “Exploration/inspection,” “Bougie,” and “Gastric band removal.”

**Table 1:** Training, validation and test datasets (hours of video)

Phase name	Training data	Validation data	Testing data
Not a phase	95.50	24.35	20.03
Ligation of short gastric vessels	70.79	18.03	13.80
Gastric transection	66.47	15.90	11.51
Bougie	5.08	1.07	0.84
Oversew staple line	42.71	13.46	6.63
Exploration/inspection	3.03	0.64	0.45
Liver retraction	1.09	0.43	0.11
Hiatal hernia repair	7.48	1.21	1.71
Gastric band removal	0.88	0.71	0.52

## Method

**Fig. 1:**
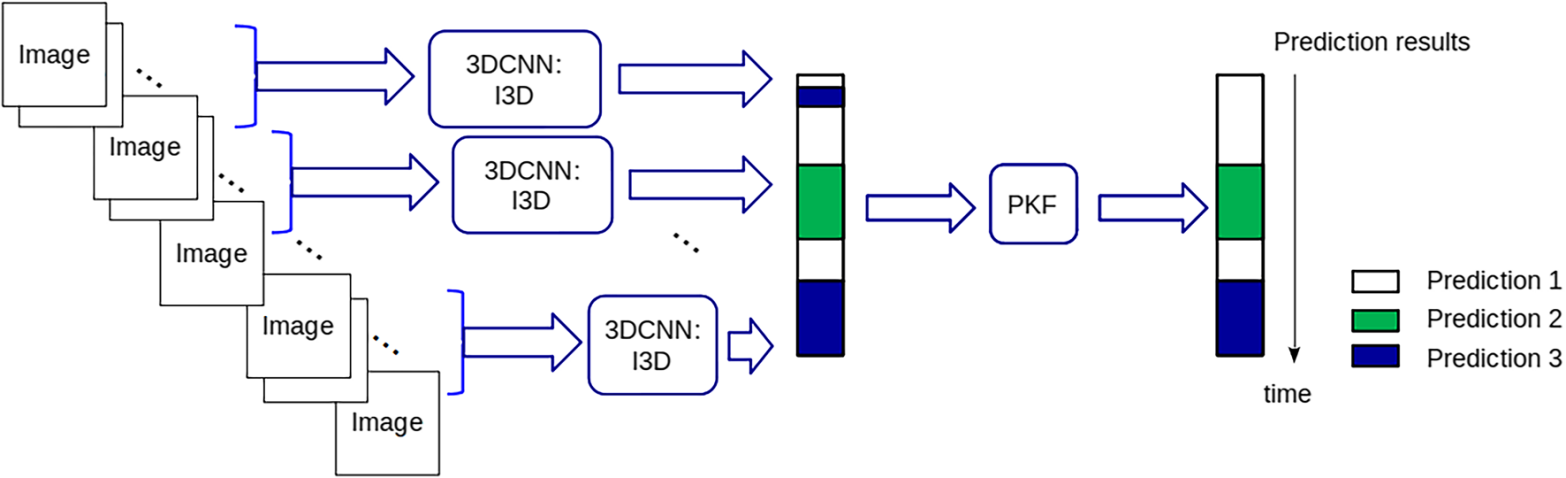
An overview of the proposed workflow: initial predictions are generated by 3D CNN from image sequences. Prior knowledge filtering is used to finalize the prediction results

The overview of our workflow is shown in Fig. 1. We divide the video into short video clips and then use the I3D architecture to make a prediction for each video clip. We concatenate the results from the video clips to obtain the initial raw prediction results for the full video. Then, we apply the prior knowledge filtering algorithm to the raw prediction results to get the finalized prediction results for the full video. The final output predictions correspond to either surgical phase predictions or not surgical phase predictions. The focal loss is used during the training to solve the imbalanced data problem and improve the prediction results.

### I3D architecture

We consider classifying each short video clip as an action recognition problem. 3DCNN has always been a typical method to facilitate spatiotemporal learning for this problem. To train a 3DCNN from scratch typically requires a large amount of training data. Carreira and Zisserman [18] proposed to inflate a 2DCNN pretrained from ImageNet along the temporal dimension to obtain a 3DCNN called inflated 3D ConvNet. Filters and pooling kernels of deep 2D ConvNets are expanded into 3D, making it possible to learn seamless spatiotemporal feature extractors from video, while leveraging successful ImageNet architectures. Carreira and Zisserman [18] trained the inflated 3D ConvNet on Kinetics human action dataset [26] to solve the action recognition problem. Inspired by their work, we adopt Inception-v1 I3D for our problem and fine-tune it on our dataset. The initial weights for Inception-v1 I3D are publicly available, we chose the RGB stream pretrained weights in this work.

### Sampling method

Synthetic minority oversampling technique (SMOTE) [27] is one of the most common ways to solve the dataset imbalance problem. We upsample the minority class and undersample the majority class to build a class-balanced dataset. For each video, each annotation segment is usually visually different. Within the same video, annotations segments labeled as “Not a phase” look different from one another. Considering the above factors, we proposed a sampling method focused on balanced sampling for each annotation segment instead of balanced sampling for each class. For each annotation segment in our video dataset, we randomly sample a fixed number of training samples. Because each annotation segment provides the same number of training samples, we named this training data sampling technique annotation segment balanced sampling (ASBS).

An example for fine-tuning I3D with ASBS is as follows: For each video, the total number of annotation segments is $n+m$, where *n* segments belong to surgical phases and *m* ($m \le n+1$) segments do not belong to any surgical phases. To fine-tune I3D on our dataset, during each training epoch, five 20-second video clips are randomly selected inside each annotation segment for each video. Sixty-four frames are sampled from each video clip as one training sample. For each training epoch, we roughly have $5v(n+m)$ training samples, where *v* is the total number of the surgical videos in the training dataset. For data augmentation purposes, we sample one frame every *a* frames when we sample 64 frames from each video clip for each training sample. The constant interval *a* is an integer and $4 \le a \le 9$.

### Focal loss

Because the duration of the surgical phase varies from each other and a large amount of the data is annotated as “Not a phase,” we have an imbalanced dataset. This class imbalance problem leads our deep learning model to achieve high prediction accuracy for the majority class and poor prediction accuracy for the minority class. Specifically, the deep learning model achieves high prediction accuracy in the “Not a phase” class, and low accuracy in the surgical phase classes. This was significantly seen in the surgical phase classes that lacked training data.

In order to solve the data imbalance problem, a new loss called focal loss [19] is proposed to tackle the foreground–background class imbalance problem for dense object detection. By reshaping the standard cross-entropy loss with a dynamically scaling factor, the loss associated with easily classifiable examples, which constitute the majority of the dataset, are down-weighted in focal loss. Because of this, focal loss gives less importance to easily classifiable examples and tends to focus on hard examples. In practice, the focal loss function is defined as

1 $$\begin{aligned} {\hbox {FL}}(p_{t}) = -\alpha (1-p_{t})^\gamma \log (p_{t}) \end{aligned}$$

where $p_{t}$ is the model’s estimated probability for the class, $\alpha$ is the balanced variant, $\gamma$ is the focusing parameter.

In focal loss, when training samples are correctly classified with a high estimated probability $p_{t}$, the value of $\gamma$ powered $1-p_{t}$ is small, and the loss for those correctly classified samples are significantly down-weighted. Their contribution to total loss is significantly reduced even if they are large in number. In contrast, when training samples are wrongly classified with a low estimated probability $p_{t}$, the loss is up-weighted. Therefore, deep learning models can focus on difficult examples that were incorrectly classified with a low estimated probability.

### Prior knowledge filtering

Most surgical videos contain frames where the surgeon is idle, frames with slight motions, frames missing important visual clues, and frames with various artifacts in the middle of the surgical phase. For such frames in a surgical video, it is hard for the deep learning model to predict accurately. Therefore, there is noise in the raw predictions from the deep learning model.

In order to filter the prediction noise, we investigate in a post-process filtering algorithm and propose the Prior Knowledge Filtering algorithm. We develop the PKF algorithm in consideration of the below aspects:

(1) Phase order: Although many surgical phases are not following a specific order, some surgical phases do follow a specific order. For example, in the sleeve gastrectomy surgical video, the “Exploration/inspection” phase happens at the beginning of the surgery. It is clear that predictions of the “Exploration/inspection” phase at the end of the surgical video are wrong predictions and need correction. We utilize our model to make predictions for the training dataset. After locating the wrong predictions in the training dataset, one option to correct these wrong predictions is to replace them with new surgical phase labels according to phase order and the model’s confidence. The other option is to correct these wrong predictions with the “Not a phase” label. We can compare our corrections with the ground truth and set up prediction correction rules. In the above-mentioned example, replacing the wrong predictions labeled as the “Exploration/inspection” phase with the “Not a phase” label can correct most of the wrong predictions on both the training and validation datasets. Therefore, we can correct those wrong predictions with the “Not a phase” label.

(2) Phase time: In order to calculate the phase time, smooth prediction results must be obtained first. A sliding window approach is used to determine the start time and the end time of each surgical phase prediction segment. We calculate the set of minimum phase time *T* with the annotation data for the training dataset. $T = \{T_{1}, T_{2}, \ldots , T_{N}\}$ where *N* is the total number of phases. For each surgical phase *i*, we set the sliding window size by

2 $$\begin{aligned} W_{i} = \min (\max (W_\mathrm{min}, \eta T_{i}), W_\mathrm{max}) \end{aligned}$$

where $W_\mathrm{min}$ is the minimum sliding window size, $W_\mathrm{max}$ is the maximum sliding window size, $\eta$ is a weighted parameter. For our specific case, we have one prediction for each second of the video. $W_\mathrm{min}$ is set to be 10, $W_\mathrm{max}$ is set to be 60, $\eta$ is set to be 0.2. We used grid search to select the parameters that allowed us to compare between the ground truth and the workflow predictions in the validation dataset.

For each surgical phase *i*, the full video prediction results are fed piece by piece to a sliding window with a length of $W_{i}$. Inside the sliding window, we count the prediction frequency value for surgical phase *i*. We set the prediction threshold value $J_{i}$ by

3 $$\begin{aligned} J_{i} = \mu _{i} W_{i} \end{aligned}$$

where $\mu _{i}$ is a weight parameter. We set $\mu _{i}$ to be 0.5 in this work.

If the prediction frequency value is greater than the prediction threshold value, the prediction result for the middle time step of the sliding window is set to be phase *i*. For adjacent predictions that share the same prediction labels, we connect them with the threshold value $L_{i}$ which we set to further solve the discontinuous prediction problem. Threshold value $L_{i}$ can be calculated by

4 $$\begin{aligned} L_{i} = \min (\nu _{i} T_{i}, L_\mathrm{max}) \end{aligned}$$

where $L_\mathrm{max}$ is the maximum connection threshold value, $\nu _{i}$ is a weight parameter. We set $L_\mathrm{max}$ to be 180 and $\nu _{i}$ to be 0.4 in this work. Here, grid search was utilized again to pick our parameters.

For each surgical phase *i*, we have smoothed prediction results. If prediction segments for different surgical phases overlap with each other, the prediction for the overlap segment is determined by the average model’s confidence calculated by

5 $$\begin{aligned} C_{i} = \frac{1}{f-e+1}\sum _{t=e}^{f}p_{(t,i)} \end{aligned}$$

where *e* is the start time step for the overlap segment, *f* is the end time step for the overlap segment, $p_{(t,i)}$ is the predicted probability at class *i* at time step *t* ($e \le t \le f$).

With the smoothed prediction result, phase time can be calculated for each surgical phase prediction segment. While many surgical phases vary in phase time, we can still correct prediction segments that are too short to be a surgical phase. We can utilize Eq. (5) to calculate the average model’s confidence for each label for those short segments. After that we can reselect labels for those short segments according to the average model’s confidence. The limitation of this approach is that it cannot filter wrong prediction phase segments that are longer than the corresponding minimum phase time. In this work, instead of utilizing the average model’s confidence, we replace those short segments with the “Not a phase” label.

(3) Phase incidence: Despite the fact that many surgical phases happen multiple times in one surgical video, some surgical phases normally only happen once or less than a fixed incidence number. We calculate the set of maximum phase incidence *I* with the annotation data for the training dataset. $I = \{I_{1}, I_{2}, \ldots , I_{N}\}$ where *N* is the total number of phases. We correct prediction segments according to phase incidence to further filter the precondition noise. For prediction segments that need corrections, we can utilize Eq. (5) to calculate the average model’s confidence for each label. We can reselect labels according to the average model’s confidence. We can locate wrong prediction segments according to the set of maximum phase incidence *I* on the validation dataset. We can further evaluate the reselect labels with the ground truth annotations. In this work, instead of utilizing the average model’s confidence, we replace those segments with the “Not a phase” label.

## Experiments

Our experiments are implemented with the Keras deep learning library using Python. Amazon EC2 P2 Instance is used for all experiments. An NVIDIA Tesla K80 GPU with 12 GB memory is used for all experiments.

### Implementation details

We utilize I3D, Focal Loss, and PKF to build our workflow and refer to it as I3D-FL-PKF. In order to quantify the improvement caused by using focal loss during the training of our deep network architecture, we also train I3D with cross-entropy loss, this baseline workflow is referred to as I3D-PKF. During the training of the I3D network, we utilize the SGD optimizer with an initial learning rate of $4e^{-3}$. We reduce the learning rate by a factor of 0.25 when there is no improvement for the validation accuracy in the last five epochs. The batch size is set to be 6. The number of epochs is set to be 50. The $\alpha$ is set to be 4 and the $\gamma$ is set to be 2 for focal loss. In order to reduce over-fitting, we utilize the dropout layer and set the dropout rate as 0.6. The input video clip length for the I3D network is 64 frames [18].

**Table 2:** Overall accuracy and weighted Jaccard score using different training techniques and different deep learning pipelines

Method	Sampling	Augmentation	Loss	PKF	Accuracy	Jaccard
C3D	ASBS	$\surd$	CE		0.7548	0.4010
C3D-PKF	ASBS	$\surd$	CE	$\surd$	0.7929	0.6591
I3D	ASBS	$\surd$	CE		0.7795	0.6506
I3D-PKF	ASBS	$\surd$	CE	$\surd$	0.8257	0.7099
I3D-PKF	SMOTE	$\surd$	CE	$\surd$	0.7892	0.6598
InceptionV3-BiLSTM-PKF	ASBS	$\surd$	CE	$\surd$	0.8078	0.6856
InceptionV3-BiLSTM-FL-PKF	ASBS	$\surd$	FL	$\surd$	0.8161	0.6989
I3D-FL-PKF	ASBS		FL	$\surd$	0.8340	0.7187
I3D-FL-PKF	ASBS	$\surd$	FL	$\surd$	0.8416	0.7327

We also conduct the data augmentation techniques following Carreira and Zisserman’s work [18] during the training process. We resize the resolution according to the smaller side of the videos to 256 pixels and randomly crop 224 * 224 patches from them. We also utilize rotation, flipping to achieve further data augmentation for the training dataset. For each training epoch, we randomly select 10% of the training samples and apply random rotation to them. The random rotation angle is randomly selected from a range of $-\,20$ degrees to 20 degrees. We also randomly select 10% of the training samples to apply random flipping. In order to quantify the improvement caused by data augmentation, we also train the I3D-FL-PKF pipeline without data augmentation for comparison.

To compare different data sampling techniques, we utilize synthetic minority oversampling technique (SMOTE) [27] to train I3D with cross-entropy loss. We upsample the minority class and undersample the majority class to build a balanced dataset. For a fair comparison, the total number of the training samples generated by SMOTE stays the same with ASBS for each training epoch.

Inception-v1-based I3D is 27 layers deep and C3D [17] is 15 layers deep. We replace I3D with C3D [17] to quantify the improvement caused by using deep 3DCNN in the pipeline. We train C3D with cross-entropy loss and refer to this workflow as C3D-PKF. We utilize the initial weights pretrained on Sports-1M Dataset for the experiments. During the training for the C3D network, we utilize the SGD optimizer with an initial learning rate of $5e^{-5}$. We reduce the learning rate by a factor of 0.25 when there is no improvement for the validation accuracy in the last five epochs. The batch size is set to be 16. The number of epochs is set to be 50. The input video clip length for the C3D network is 16 frames [17]. For a fair comparison, we conduct similar data augmentation techniques during the training for the C3D workflow.

In order to quantify the improvement caused by using the I3D as the deep network architecture, a similar CNN–RNN workflow was implemented with InceptionV3-BiLSTM as a replacement for I3D. We select InceptionV3 instead of ResNet [14, 16] as the CNN because it performs better on multiple datasets in fine-tuning experiments [28]. Very similarly to Hirenkumar’s approach [13], the final classification layer in InceptionV3 was removed, and 2048-dimensional feature vectors were extracted from InceptionV3 with the global average pooling layer (GAP). We utilize single-layered bidirectional LSTM (BiLSTM) with 256 hidden neurons to capture the temporal information with those extracted features. The baseline workflow is referred to as InceptionV3-BiLSTM-FL-PKF when we train the deep network architecture with focal loss and is referred to as InceptionV3-BiLSTM-PKF when we train the deep network architecture with the cross-entropy loss. During the training for the InceptionV3-BiLSTM network, we utilize the SGD optimizer with an initial learning rate of $1e^{-4}$. Same as training the I3D network, the learning rate is reduced by a factor of 0.25 when there is no improvement for the validation accuracy in the last five epochs. Similar to training the I3D network, we utilize dropout and the data augmentation techniques during the training for our CNN–RNN workflow. The input video clip length for the InceptionV3-BiLSTM network is set to be 20 frames due to limited computational memory. For a fair comparison, we conduct end-to-end training for our CNN–RNN networks. The batch size is set to be 6. The number of epochs is set to be 50.

### Results

Table 2 shows the overall accuracy and weighted Jaccard score for experiments conducted on our test dataset. C3D-PKF outperforms C3D when we train the models with ASBS and cross-entropy loss. I3D-PKF also outperforms I3D. Those results demonstrate that PKF can improve performance. I3D outperforms C3D by around 2.5% from the accuracy aspect which demonstrates the importance of utilizing deep 3DCNN in the workflow. From the data sampling aspect, I3D-PKF trained by our ASBS technique outperforms I3D-PKF trained by SMOTE. Our data sampling technique ASBS is more suitable for our use case. Results demonstrate our ASBS can alleviate the data imbalanced problem. The accuracy of our InceptionV3-BiLSTM-PKF workflow is 0.8078, and the weighted Jaccard score is 0.6856. Similar to the previous research [17], our shallow C3D workflow does not outperform the CNN–RNN workflow. This demonstrates the limits of utilizing shallow 3DCNN to capture spatial and temporal information for surgical phase recognition. The accuracy of our I3D-PKF workflow is 0.8257, and the weighted Jaccard score is 0.7099. The accuracy of our InceptionV3-BiLSTM-FL-PKF workflow is 0.8161, and the weighted Jaccard score is 0.6989. The accuracy of our I3D-FL-PKF workflow is 0.8416, and the weighted Jaccard score is 0.7327. Results show that our 3DCNN workflow outperforms CNN–RNN workflow, and networks trained with focal loss outperform networks trained with cross-entropy loss. From the data augmentation aspect, data augmentation can improve accuracy and the weighted Jaccard score for our proposed workflow.

Table 3 shows our I3D-FL-PKF workflow pipeline performance in detail where we calculate precision, recall, and F1-score for each surgical phase. From Table 3, our deep learning model performs well in several surgical phases like “Oversew staple line,” “Ligation of short gastric vessels,” “Gastric transection,” and so on. The F1-scores for those phases are over or equal to 0.88. Our deep learning model does not perform well in surgical phases like “Exploration/inspection,” “Liver retraction,” and “Bougie.” The F1-scores for those surgical phases are less than or equal to 0.32. As shown in Table 1, the reason why those surgical phases do not perform well is likely due to the lack of training data.

**Table 3:** Detailed performance for the I3D-FL-PKF workflow pipeline

Phase name	Precision	Recall	F1 score
Not a phase	0.80	0.75	0.78
Ligation of short gastric vessels	0.86	0.93	0.89
Gastric transection	0.89	0.90	0.90
Bougie	0.35	0.30	0.32
Oversew staple line	0.86	0.94	0.90
Exploration/inspection	0.74	0.17	0.28
Liver retraction	0.18	0.68	0.28
Hiatal hernia repair	0.85	0.92	0.88
Gastric band removal	0.85	0.69	0.76

**Fig. 2:**
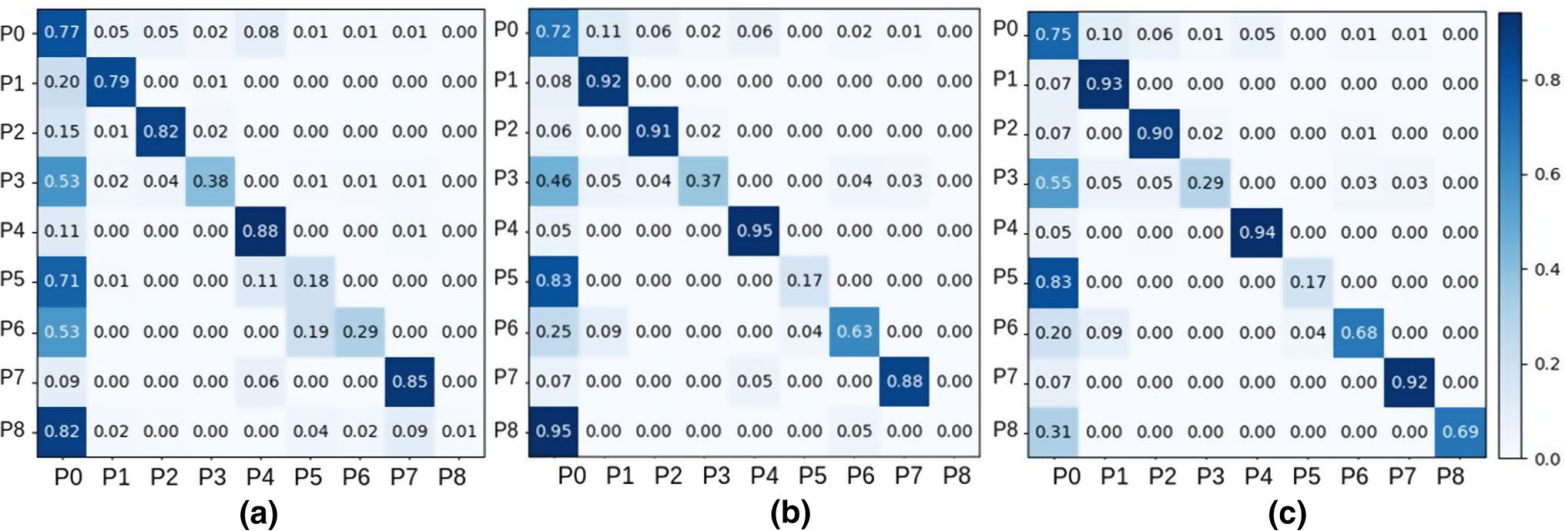
Confusion matrices for phase recognition results: **a** I3D prediction results, **b** I3D-PKF prediction results, **c** I3D-FL-PKF prediction results. The *X* and *Y*-axis represent predicted label and ground truth, respectively. The “Not a phase” is denoted as P0. The “Ligation of short gastric vessels” phase is denoted as P1. The “Gastric transection” phase is denoted as P2. The “Bougie” phase is denoted as P3. The “Oversew staple line” phase is denoted as P4. The “Exploration/inspection” phase is denoted as P5. The “Liver retraction” phase is denoted as P6. The “Hiatal hernia repair” phase is denoted as P7. The “Gastric band removal” phase is denoted as P8

**Fig. 3:**
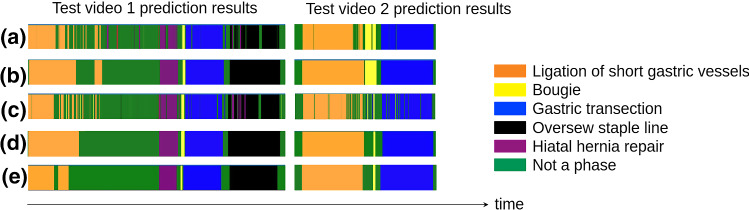
Color-coded ribbon illustration for phase recognition results: **a** InceptionV3-BiLSTM-FL prediction results, **b** InceptionV3-BiLSTM-FL-PKF prediction results, **c** I3D-FL prediction results, **d** I3D-FL-PKF prediction results, **e** Ground Truth

In order to have a better understanding of the model performance, we plot the confusion matrices in Fig. 2. Adding PKF in the workflow can improve sensitivity for five surgical phases including “Ligation of short gastric vessels,” “Oversew staple line,” “Gastric transection,” and so on. Utilizing focal loss during training can help improve sensitivity for “Liver retraction,” “Hiatal hernia repair,” and “Gastric band removal.” As shown in Table 1, we lack training data for those surgical phases. This demonstrates that using focal loss during training can alleviate the imbalanced data problem. Surgical phases are misclassified as “Not a phase” in most prediction errors. This is because some video clips annotated as “Not a phase” do not have easily distinguishable visual clues. “Not a phase” class includes many surgical activities which make it hard for the model to learn well. The “Exploration/inspection” surgical phase is hard for the model to learn well. The reason might be that video clips annotated as “Exploration/inspection” look similar to some video clips annotated as “Not a phase.” The “Bougie” surgical phase is also hard for the model to learn well. This is likely due to its short duration. Both focal loss and PKF fail to improve the performance in the “Exploration/inspection” phase and the “Bougie” phase.

As shown in Fig. 3, we visualize the raw prediction results from I3D-FL model output and InceptionV3-BiLSTM-FL model output for two test videos as examples as well as visualize the predictions from I3D-FL-PKF workflow and InceptionV3-BiLSTM-FL-PKF workflow for those two test videos. The I3D-FL-PKF workflow output results clearly have less prediction noise than the InceptionV3-BiLSTM-FL-PKF workflow output. The results demonstrate the ability of the I3D-FL-PKF workflow to obtain consistent and smooth predictions both within the surgical phases and during the phase transitions.

## Conclusion

In this paper, we implement a 3DCNN-based surgical workflow recognition pipeline named I3D-FL-PKF and apply it to sleeve gastrectomy surgical workflow recognition. Results show that utilizing focal loss, the prior knowledge filtering, our proposed annotation segment balanced sampling technique, and the data augmentation technique can improve the performance of the pipeline. By utilizing 3DCNN, focal loss, and the prior knowledge filtering, the proposed workflow outperforms the traditional CNN–RNN design. It can obtain consistent and smooth predictions both within the surgical phases and during the phase transitions. For future work, we intend to apply the proposed approach to other surgeries like radical retropubic prostatectomy, sacrocolpopexy, and ventral hernia repair for surgical workflow recognition. We want to design a weighted focal loss that focuses on the surgical phases with lower performance. To replace the grid search method, we want to design a better hyperparameter selection method for the prior knowledge filtering algorithm. In the future, we also want to apply the proposed approach to other vision-based projects, such as detecting surgical tool usage in surgical videos and identifying when the surgeon is idle during the surgery.
